# EchoAGE: Echocardiography-based Neural Network Model Forecasting Heart Biological Age

**DOI:** 10.14336/AD.2024.0615

**Published:** 2025-07-28

**Authors:** Anastasia A. Kobelyatskaya, Zulfiya G. Guvatova, Olga N. Tkacheva, Fedor I. Isaev, Anastasiia L. Kungurtseva, Alisa V. Vitebskaya, Anna V. Kudryavtseva, Ekaterina V. Plokhova, Lubov V. Machekhina, Irina D. Strazhesko, Alexey A. Moskalev

**Affiliations:** ^1^Russian Clinical Research Center for Gerontology, Pirogov Russian National Research Medical University, Ministry of Healthcare of the Russian Federation, Moscow 129226, Russia.; ^2^Engelhardt Institute of Molecular Biology, Russian Academy of Sciences, Moscow 119991, Russia.; ^3^Kivach Clinic, 186202 Konchezero, Russia; ^4^Pediatric Endocrinology Department, I.M. Sechenov First Moscow State Medical University, 119991 Moscow, Russia

**Keywords:** biological age, echocardiography, neural network

## Abstract

Biological age is a personalized measure of the health status of an organism, organ, or system, as opposed to simply accounting for chronological age. To date, there have been known attempts to create estimators of biological age based on various biomedical data. In this work, we focused on developing an approach for assessing heart biological age using echocardiographic data. The current study included echocardiographic data from more than 5,000 different cases. As a result, we created EchoAGE - neural network model to determine heart biological age, that was tested on echocardiographic data from patients with age-related diseases, patients with multimorbidity, children with progeria syndrome, and diachronic data series. The model estimates biological age with a Mean Absolute Error of approximately 3.5 years, an R-squared value of around 0.88, and a Spearman's rank correlation coefficient greater than 0.9 in men and women. EchoAGE uses indicators such as E/A ratio of maximum flow rates in the first and second phases, thicknesses of the interventricular septum and the posterior left ventricular wall, cardiac output, and relative wall thickness. In addition, we have applied an AI explanation algorithm to improve understanding of how the model performs an assessment.

## INTRODUCTION

It is known that chronological age may differ from biological age. Unlike chronological age, which is quite easy to determine, biological age more likely corresponds to the state of health, according to a combined assessment of morphological, physiological, and biochemical states in many human body systems. By calculating the biological age, it is possible to detect the acceleration of aging before a certain pathological condition occurs [1-2]. Biological age includes both a genetic and an ontological component, taking into account a person's lifestyle and the deterioration of their body. Calculating the biological age is a more complex, yet personalized, approach to assessing an organism's state compared to chronological age. There is still no single formula for determining a person's biological age, but attempts have been made to create local estimates. These estimates are usually based on the state of an organ or system in a person's body. The most common methods for determining biological age use general functional and anthropometric indicators, as well as epigenetic markers [3], and morphofunctional assessments of specific functions in an organism or an organ [4-6]. Common combinations of these indicators include general parameters (such as height, weight, and body mass index) combined with more local indicators, such as genetic components or narrowly focused assessments of a specific organ or system.

One such local assessment may be a study of a patient's cardiovascular system, determining its biological age, and attempting to correct it by minimizing factors associated with it [4]. Probably, such an approach would help reduce the incidence of major age-related diseases, including diabetes mellitus (E10-E14, by ICD 10th), hypertension (I10-I15), heart disease (I21-I25), and stroke (I63) [4; 7].

To date, several methods are known for determining the biological age of the cardiovascular system. Models have been created based on electrocardiogram (ECG) indicators [4; 8], heart rate [9], tonometry, and in combination with biochemical markers [7; 10-13]. It is important to note that echocardiography is one of the most informative instrumental methods for diagnosing cardiovascular problems. During echocardiography, morphological and functional changes in the heart are assessed, and indicators reflecting the size of walls, chamber volumes, and blood flow rates are calculated, etc.

Most of the current approaches are implemented using linear or logistic regression. These machine learning methods are widely used in biomedical classification and regression tasks. However, there are more advanced algorithms such as random forests, boosting, and neural networks [14]. Of these modern techniques, neural network algorithms have a more complex and flexible configuration [15]. The advantage of these methods is that they do not require us to determine the nature of the dependencies beforehand. If the dependence is already encoded in the data, these methods are likely to be better at describing it than regression techniques. At the same time, there is a point of view that these techniques are black boxes, meaning there is no clear and understandable human formula by which the evaluation takes place. Until recently, this concerned many experts who are accustomed to interpreting formulas with explicit coefficients. However, today we have algorithms for post-processing a neural network's solution - explainable artificial intelligence (XAI), which explains the estimates produced by a neural network and turns a black box into a white box, allowing researchers to understand why an estimate is one way or the other [16].

The purpose of our work was to create, train, and test a predictive model using a neural network algorithm to estimate biological age based on echocardiography data.

The results demonstrate that echocardiographic assessments can be used to determine the biological heart age. Consequently, we created a model for determining the age of a human heart based on echocardiography data. Additionally, using our approach, we estimated the ages of more than 5,000 cases, including those with age-related conditions and children with progeria syndrome.

## MATERIALS AND METHODS

### Cohorts

The present study includes pseudonymized echocardiography data from 5,253 cases from Caucasian patients. The echocardiography data from 5,253 cases observed in several medical departments formed into five different cohorts. Two of these cohorts were patients at the Russian Clinical Research Center for Gerontology (Pirogov Russian National Research Medical University) and two cohorts includes patients from the Kivach Clinic. The last cohort consisted of children with Hutchinson-Gilford Syndrome separately obtained from Pediatric Endocrinology Department (I.M. Sechenov First Moscow State Medical University, Table 1). Among these 5,253 cases there are both patients with no age-associated diseases, as well as with cardiovascular diseases, endocrine, metabolic and digestive disorders, and also multimorbidity patients and children with Progeria. Cohorts A and B included patients without any age-related diseases, amounting to 1,064 cases. These two cohorts were used to develop, train, and validate the model. The remaining 4,189 cases were accompanied by various conditions and were used as an external independent dataset to test the model and analyze the relationship between the assessment model and diseases.

**Table 1:** Characteristics of the studied cohorts.

		Cohort A	Cohort B	Cohort C	Cohort D	Cohort E
**Cases, n**		242	822	93	4092	4
Sex, n	Male	78	454	43	2329	2
	**Female**	169	368	50	1763	2
**Age, mean (min-max)**		50 (23-91)	47 (5-83)	67 (39-91)	49 (4-86)	6 (4-8)
**Patients with age-related diseases, yes / no**		no	no	yes	yes	yes
**Contribution in model**		B	B	P	P	P

Note: B - build, P - approbation.

### Receiving echocardiographic data

Transthoracic echocardiography was performed using an iE-33 S5-1 ultrasound device (PHILIPS Medical Systems), Vivid E9 6S-D and Vivid E9 M5S ultrasound devices (GE Healthcare). Linear dimensions were estimated from images obtained in the parasternal position along the long axis of the left ventricle, in B-mode and M-mode. Volumetric indicators were obtained in two-dimensional mode, by tracing the boundaries of the endocardium, in the apical 4 chamber and 2 chamber positions, (biplane disk method), into the systole (end-systolic volume) and diastole (end-diastolic volume), respectively [17]. The relative thickness of the walls of the left ventricular was calculated according to the following formula: RWT = (thickness of interventricular septum + posterior wall thickness) / end-diastolic left ventricular diameter [17]. Fractional shortening, ejection fraction, and stroke volume were measured to evaluate systolic function in the left ventricles. The fractional shortening (%) was calculated using the formula: (end-diastolic left ventricular diameter - end-systolic left ventricular diameter)/ end-diastolic left ventricular diameter) x 100. The ejection fraction (%) was calculated by disk method (Modified Simpson) in B-mode. Cardiac Output or Minute Heart Volume (L/minute) according to formula: Stroke Volume x Heart Rate. The Cardiac Index was obtained by ratio of Cardiac Output to Body Surface Area (L/minute/m^2). Stroke Volume (ml) defined as difference between end-diastolic volume and end-systolic volume. Doppler study of transmitral flow in apical 4 chamber position performed. Measured: E/A Ratio (peak velocities A and E waves), DT (deceleration time), and IVRT (isovolumic relaxation time) [18].

### Data analysis

The statistical analysis was conducted in the statistical software environment of R (www.r-project.org). We have excluded cases with incomplete data. The studied echocardiogram parameters, like most medical data, did not have a normal distribution. Indicators with unnormalized body size scores (cm, mm, L, and ml) were normalized to patient height (cm). Spearman's rank correlation analysis was used as a nonparametric test (cor.test function, rdrr.io/r/stats/cor.test.html.) to calculate the strength of the relationship between variables (echocardiography estimates) and age. Three correlation analysis modes were performed: one for the entire cohort, one for men only, and one for women only. Echocardiographic indicators with the strongest relationship to age (rho ≥ 0.6 and p-value ≤ 0.05) were chosen as predictors. Before creating and training the model, the Cohort A was divided into training and testing datasets (2:1). The principal component analysis was performed using prcomp from the stat library (www.r-project.org).

As a model, the architecture of a fully connected neural network (CNN) was created using Keras (https://keras.io) and Tensorflow libraries (https://tensorflow.rstudio.com). The architecture of the model is a deep network (DNN) containing 10 hidden layers. Echocardiography estimates (combinations of selected predictors) were used as an input layer. Each hidden layer consisted of 50-750 neurons with swish activation function. The output layer was a single neuron with a relu activation function. MSE was used as the error function. The adam algorithm with the parameter learning rate = 0.003 was used as an optimizer (https://arxiv.org/abs/1412.6980). To prevent overfitting, the model was subject to preservation after passing each epoch, the number of epochs giving the maximum quality of the model was considered optimal (the maximum number of epochs = 200).

The randomForest [19] and xgboost [20] libraries were used to build models using Random forests and XGBoost algorithms.

The metrics for model quality during training are MAE (Mean Absolute Error), MSE (Mean Square Error), MedAE (Median absolute error), RMSE (Root Mean Square Error), R2 (Determination Coefficient), and ε-acc (ε-accuracy, or epsilon-accuracy, where ε = 10, i.e., ±10 years is the accuracy spread). These metrics were calculated using the caret R package (https://github.com/topepo/caret/). The most accurate models were combined into a final two models for each sex.

As a post-processing of the created models, we used the explicable artificial intelligence (XAI) - SHapley Additive exPlanations (SHAP) algorithm to explain the estimates obtained by the model, the kernelshap (https://cran.r-universe.dev/kernelshap) and shapviz (https://github.com/cran/shapviz) libraries.

The frequency analysis was performed using the Pearson's chi-squared statistical test, using the chisq.test function in R.

Visualizations of the obtained results were performed using the ggplot2 package (https://ggplot2.tidyverse.org/).

## RESULTS

### Study design

**Figure 1.**
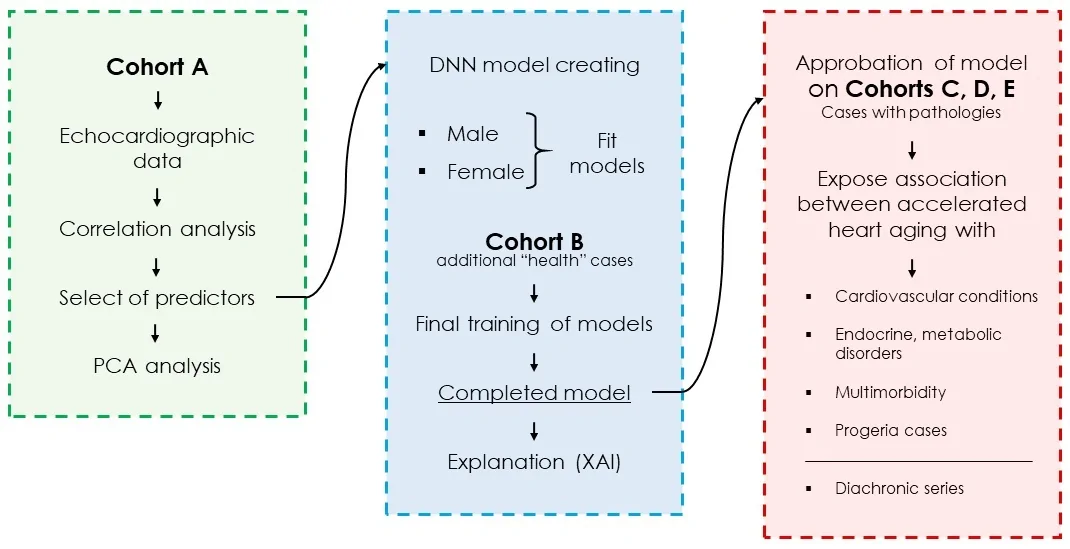
Experimental design flowchart of EchoAGE study.

In our work, we proposed a neural network as a model for determining the biological heart age. We used echocardiography parameters as input data and selected the most promising predictors based on results of correlation analysis. Using these predictors, we created and trained a model using data from patients without age-related diseases. Finally, we verified the work of our model using data from various cases (Fig. 1).

### Determining predictors

**Figure 2.**
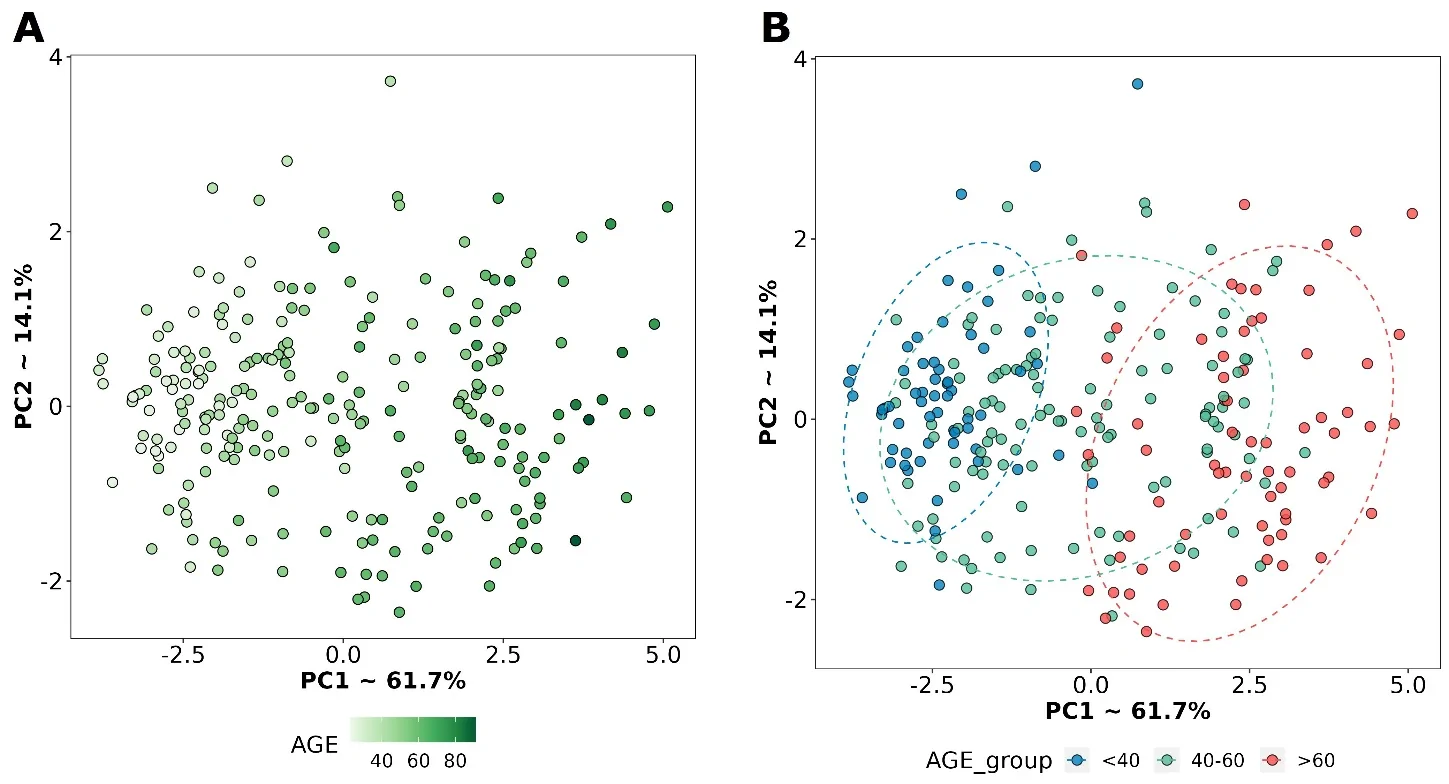
**PCA plot based on selected predictors**. (**A**) Continuous age view (green gradient); (B) three age groups view (<40 - blue, 40-60 - green, >60 - red).

The first stage was an assessment of the ability of echocardiography data to describe human age. For this purpose, a nonparametric correlation analysis was performed on all Cohort A as well as on groups of men and women separately. Out of 45 indicators, the top 15 were identified which demonstrated a maximum correlation with age greater than 0.6 with a *p*-value <0.05 (Supplementary Table 1). Among these, there are indicators that are not included in the most common investigation protocols, and also there are parameters that represent binary factor transformations of the original values. In this study, we focused on parameters with a greater predictive potential that are essential during echocardiography and maintain a gradient of dependency. As a result, we selected five candidates for predictors: EA - E/A ratio of maximum flow rates in the first and second phases, IVS - thickness of the interventricular septum, LVPW - thickness of the posterior left ventricular wall, LVCO - cardiac output, and RWT - relative wall thickness (Table 2). Based on these parameters, we conducted an analysis of the principal components (PCA). The results of the first two components clearly demonstrate the relationship between the aggregate of these parameters and age, both as a continuous variable (Fig.2A) and in age groups (Fig.2B).

**Table 2:** Correlations of selected predictors with age.

ID	All cases		Male		Female	

	rho	*p*-value	rho	*p*-value	rho	*p*-value
RWT	0.77	7.6×10^-51^	0.76	7.9×10^-16^	0.81	5.1×10^-40^
IVS	0.66	4.2×10^-32^	0.54	2.4×10^-07^	0.70	2.5×10^-26^
LVPW	0.62	2.0×10^-27^	0.57	3.4×10^-08^	0.64	1.8×10^-20^
EA	-0.68	5.6×10^-35^	-0.64	2.7×10^-10^	-0.73	1.1×10^-29^
LVCO	-0.74	1.5×10^-44^	-0.71	2.6×10^-13^	-0.72	9.1×10^-29^

Note: rho (Spearman correlation coefficient), LVCO (cardiac output, L/minute), EA (E/A ratio of maximum flow rates in the first and second phases), RWT (relative wall thickness), IVS (thickness of the interventricular septum, cm), LVPW (thickness of the posterior left ventricular wall, cm).

### Creating a predictive model

Based on the selected five predictors, we identified combinations to train and test the model predicting age, which serves as the input layer in the neural network architecture. After completion of testing, we noticed that the most reasonable combination is the use of all five predictors (Supplementary Table 2-1).

In addition, we tested this combination of predictors using other common modern machine learning algorithms, such as Random Forest and XGBoost, which are inferior to neural network algorithms (Supplementary Table 2-2).

**Table 3:** Quality indicators of the final model.

Data set	MSE	MAE	MedAE	RMSE	R^2^	rho	*ε*-acc5	*ε*-acc10
**Both sexes**								
**Cohort A Train**	20.54	3.26	2.00	4.53	0.90	0.95	0.85	0.97
**Cohort A Test**	19.19	3.05	2.00	4.38	0.90	0.95	0.84	0.96
**Cohort B**	25.34	3.64	3.00	5.03	0.85	0.92	0.76	0.95
**Cohort B children**	5.57	1.38	1.00	2.36	0.76	0.87	0.95	1.00
**Males**								
**Cohort A Train**	19.43	3.09	2.00	4.41	0.87	0.93	0.89	0.96
**Cohort A Test**	14.96	2.62	2.00	3.87	0.92	0.96	0.88	0.96
**Cohort B**	27.02	3.78	3.00	5.20	0.82	0.91	0.74	0.94
**Cohort B children**	7.70	1.50	0.50	2.77	0.65	0.81	0.90	1.00
**Females**								
**Cohort A Train**	21.09	3.35	3.00	4.59	0.91	0.96	0.83	0.97
**Cohort A Test**	20.92	3.22	2.00	4.57	0.90	0.95	0.83	0.97
**Cohort B**	23.26	3.48	3.00	4.82	0.88	0.94	0.78	0.96
**Cohort B children**	3.64	1.27	1.00	1.91	0.85	0.92	1.00	1.00

Note: Cohort A Train - train set, Cohort A Test - test set, Cohort B - additional health set, Cohort B children - cases with age < 18 years. MSE (Mean Square Error), MAE (Mean Absolute Error), MedAE (Median absolute error), RMSE (Root Mean Square Error), R^2^ (Determination Coefficient), rho (Spearman correlation coefficient), and *ε*-acc (*ε*-precision or epsilon precision, where *ε* = 5 or *ε* = 10, i.e. ±5 or ± 10 years is the spread when evaluating accuracy).

Since the correlation between the selected parameters varies depending on sex, further training was carried out in two modes - separately for men and women. The most optimal architectural solution was to create pre-trained models of a multi-channel architecture for each sex. At this stage of the work, we have another dataset. At this stage of the work, we have received additional extensive dataset (Cohort B), which includes echocardiographic data of patients who also do not have cardiovascular and endocrine pathologies (including children and young persons). Cohort B was used in final training of the model.

**Figure 3.**
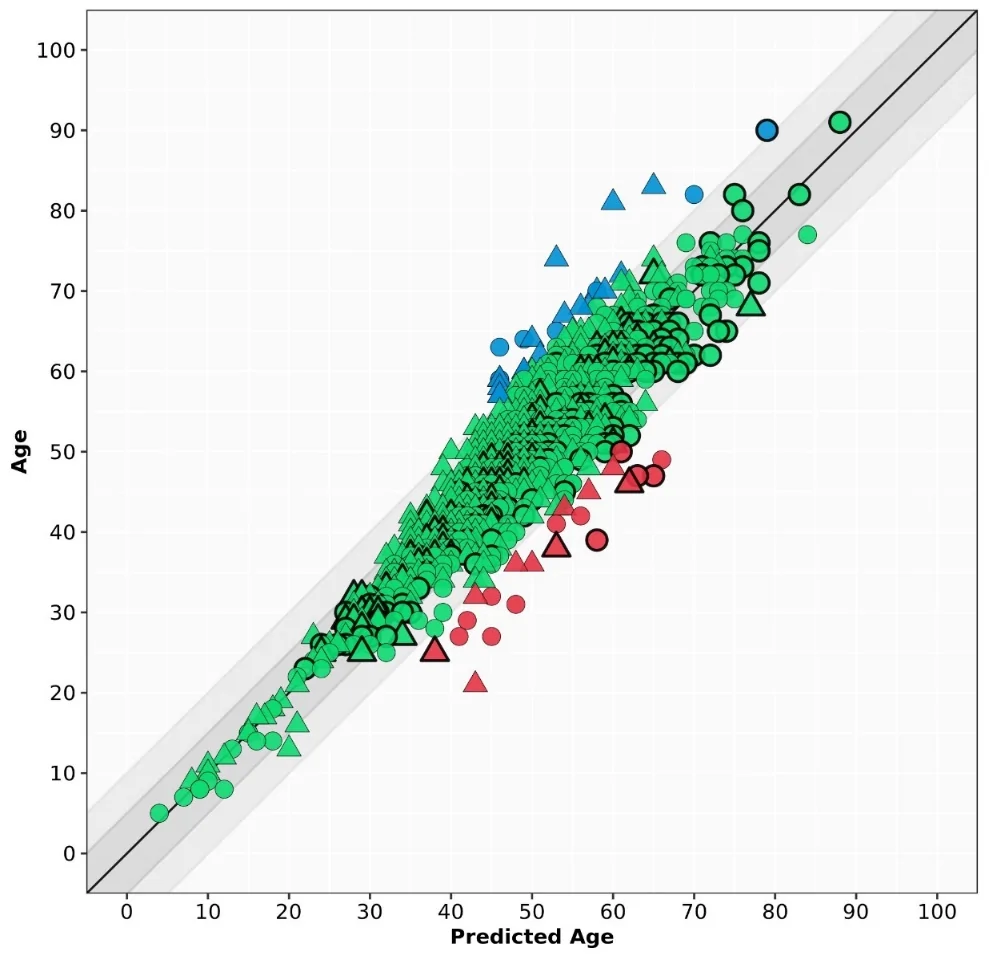
**The results of the work of final neural network model**. Black outlined - cases of initial Cohort A, without outlined - additional Cohort B. Green - cases in the 10-year spread, blue - cases below the 10-year difference, red - above the 10-year difference, triangle - men, circle - women. The X-axis is the predicted age, and the Y-axis is the real age

### The model's operation results

Eventually predictive age model provide five echocardiogram estimates as input data: LVCO (cardiac output, l/min), EA (E/A, ratio of maximum flow rates in the 1st and 2nd phases), RWT (ratio of wall thickness), IVS (thickness of the interventricular septum, cm), LVPW (thickness of the posterior wall of the left ventricle, cm), and also height (cm) and sex of the subject.

The final model, designed to predict age, has high-quality indicators for both the training and verification datasets (Table 3).

Of the 1,069 conditionally healthy cases (Cohorts A and B), only 48 (less 5%) had a predicted age exceeding the spread of ± 10 years from the real one (abs delta > 10), where 26 had a negative delta (delta < -10 years) and 22 had a positive delta (delta > 10 years, Fig.3).

### Explanation of the Final Model's Operation

**Figure 4.**
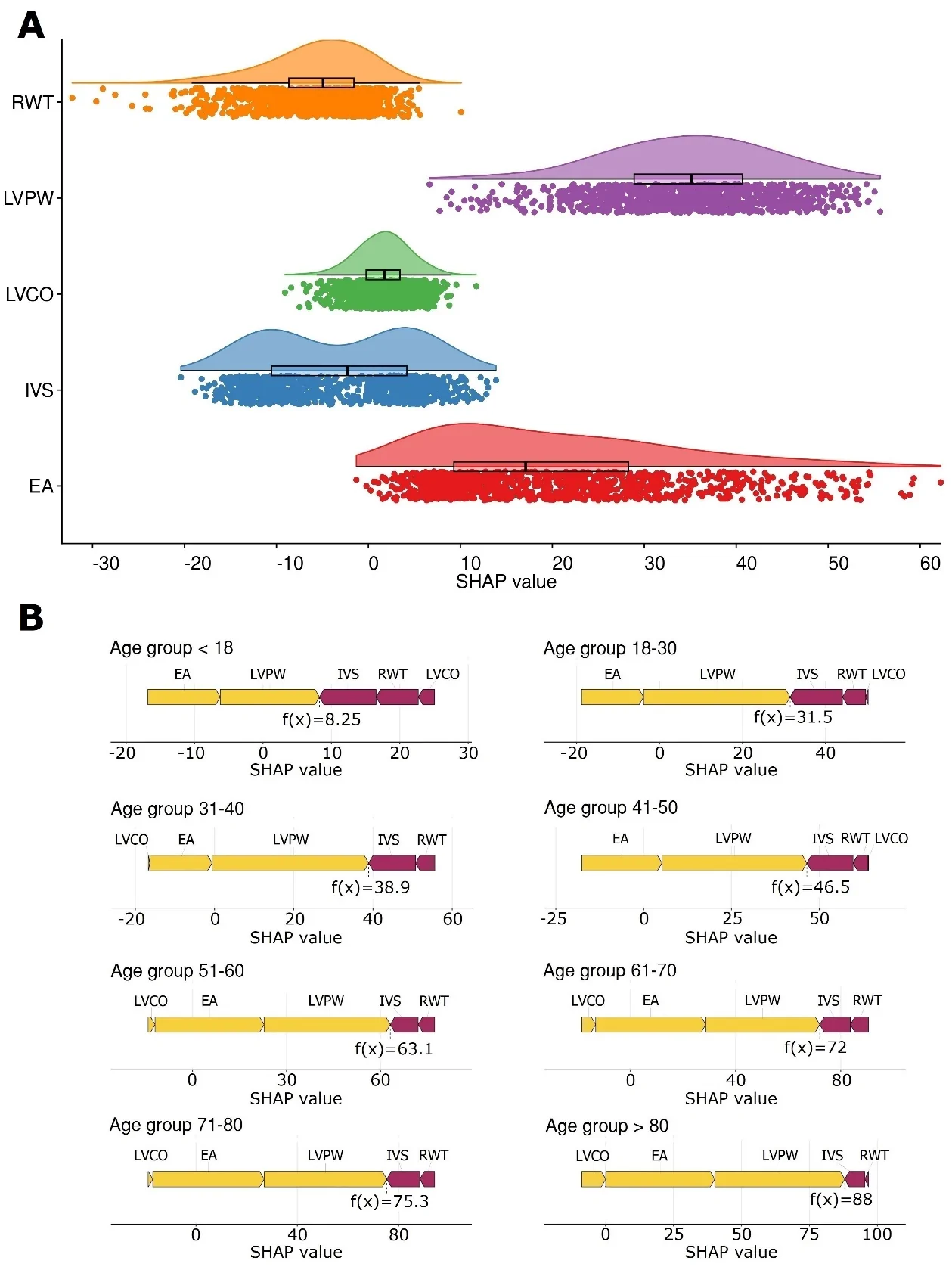
**XAI results**. Violin plot of SHAP values (in years) distribution for each predictor (A). Collapsed SHAP values (in years) in age groups (B).

For a detailed understanding of the age assessment model, we applied an AI-based post-processing and explanation algorithm (XAI) that allowed us to transform what is known as a "black box" into a "white box". The SHapley Additive explanations (SHAP) method was used as an explanation tool, which allows users to determine the contribution of each predictor to the overall assessment by calculating SHAP values (expressed in years) for each predictor within each individual case (Fig.4A). The SHAP values indicate the contribution of each item to the forecast. They allow us to gain a comprehensive understanding of the model's prediction from both a local and global perspective.

When considering individual age groups, it can be noted that the LVPW indicator has the highest SHAP values (in years), which is always positive, and, apparently, serves as the basis for the assessment made by the model for all ages (Fig.4B). Besides, the EA indicator also has a large positive SHAP value, although it can be negative. The other three predictors (IVS, LVCO, and RWT) serve as corrective ones, determining the final individual assessment. Moreover, it should be noted that their contribution direction changes with age: in young people, they usually have negative SHAP values, but as the age group increases, they acquire positive values, i.e. they add years to the assessment.

### Approbation of the final model

In addition to the above-described datasets of conditionally healthy cases (Cohorts A, B), we had the opportunity to test the model on other data from extremely diverse cases (Cohorts C, D, E). We analyzed the data of these cohorts using the developed model (Table 4).

**Table 4:** Quality indicators of the final model for additional cohorts.

Dataset	MSE	MAE	MedAE	RMSE	R^2^	rho	*ε*-acc5	*ε*-acc10
**Both sexes**								
**Cohort C**	140.44	9.75	9.0	11.85	0.50	0.70	0.31	0.57
**Cohort D**	119.76	8.39	7.0	10.94	0.40	0.64	0.42	0.70
**Cohort E**	1620.75	40.25	40.5	40.26	0.69	0.83	0.00	0.00
**Males**								
**Cohort C**	151.69	10.29	10.0	12.32	0.51	0.72	0.27	0.53
**Cohort D**	116.00	8.27	7.0	10.77	0.36	0.60	0.42	0.70
**Cohort E**	1640.50	40.50	40.5	40.50	1.00	1.00	0.00	0.00
**Females**								
**Cohort C**	107.53	8.99	9.0	10.37	0.56	0.75	0.29	0.58
**Cohort D**	124.72	8.55	7.0	11.17	0.45	0.67	0.42	0.69
**Cohort E**	1601.00	40.00	40.0	40.01	1.00	1.00	0.00	0.00

Note: Cohort A Train - train set, Cohort A Test - test set, Cohort B - additional health set, Cohort B children - cases with age < 18 years. MSE (Mean Square Error), MAE (Mean Absolute Error), MedAE (Median absolute error), RMSE (Root Mean Square Error), R^2^ (Determination Coefficient), rho (Spearman correlation coefficient), and *ε*-acc (*ε*-precision or epsilon precision, where *ε* = 5 or *ε* = 10, i.e. ±5 or ± 10 years is the spread when evaluating accuracy).

Cohort E included four cases of progeria: two boys and two girls ages 4-8 years. The predicted age was approximately 45 for everyone.

According to the results of the algorithm used to explain the model, it is possible to assume that, as a result of training, the weights in the model have been selected such that the LVPW predictor acts as the main support. It gives a strictly positive evaluation (i.e., it does not deduct years), presumably due to this the model adds years in some excess, and at the expense of other predictors, corrects its overall evaluation.

### The association between accelerated ageing of the heart and pathological conditions

The difference between the predicted age and the actual one was indicated by the delta (Δ). To assess the association between Δ and various pathologies, we divided the all cohorts into three groups (called Δ groups): PositiveΔ10 (where Δ > 10 years); NegativeΔ10 where Δ < -10 years; and NeutralΔ when the estimate does not exceed 10-year spread. It is important to note that the studied cohort included children’s data (real age less than 18 years) and the 10-year spread is too large for them. Therefore, only for this age group, the spread was ±5 years. Additionally, we identified the main age groups: less than 18 years, 18-30, 31-40, 41-50, 51-60, 61-70, 71-80, >80. Then, for each of the Δ groups that crossing with the age groups, we calculated the frequency of a specific disease and compared the frequencies obtained in the projection of the Δ group to determine which diseases are associated with not age itself (it is already known), and accelerated heart aging, which is observed in patients with PositiveΔ10. We were focused on the frequency differences between PosΔ10 and NegΔ10, as well as between PosΔ10 and NeutralΔ. In this part of our work, we examined cardiovascular diseases, endocrine pathologies, metabolic and digestive disorders. Additionally, we separately analyzed multimorbid cases and children with progeria. Finally, some patients had repeated evaluations with intervals of about a year, and we conducted analysis in a diachronic manner.

**Figure 5.**
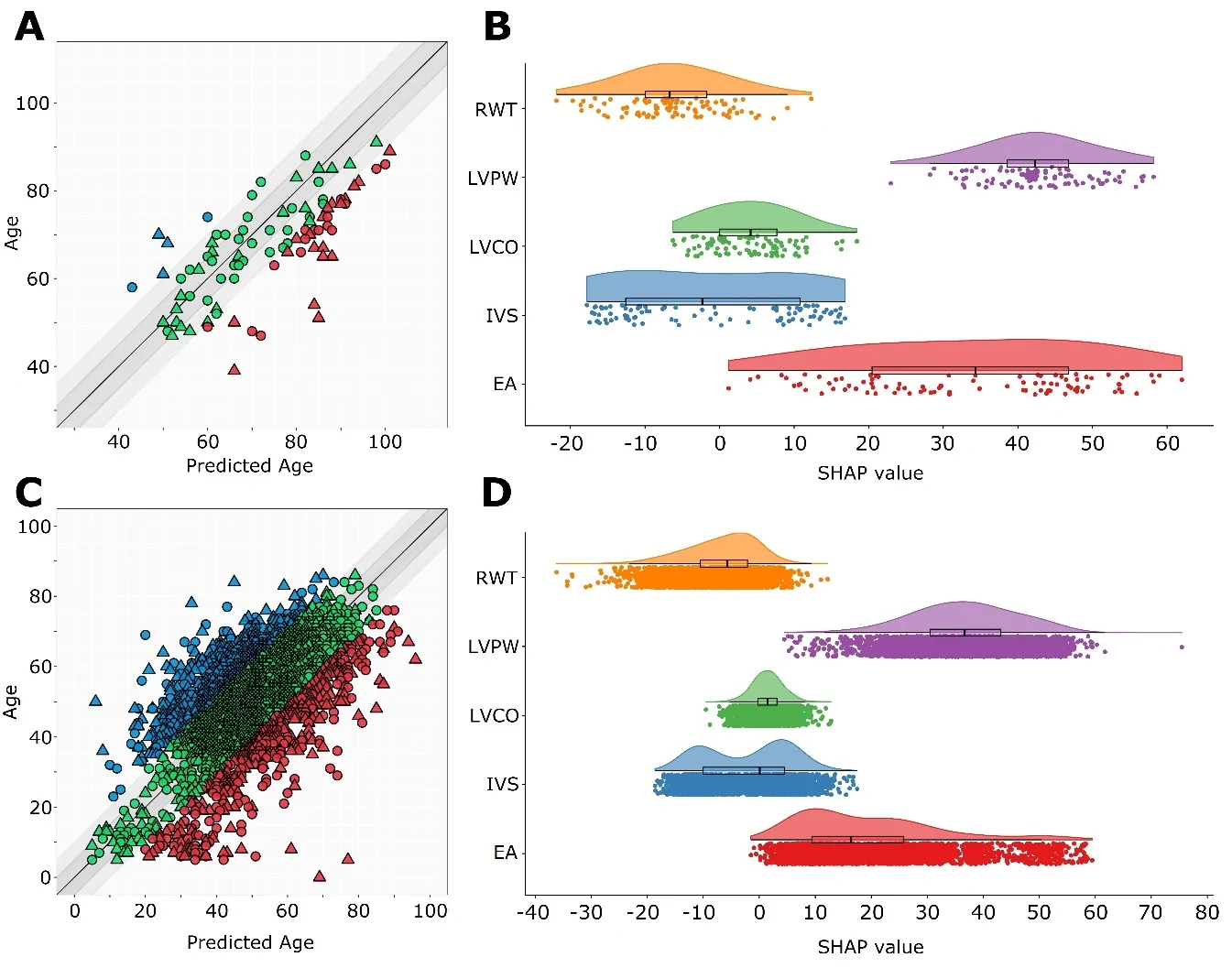
**The results of the work of final neural network model in cohort C (A panel), cohort D (C panel)**. Green - cases in the 10-year spread, blue - cases below the 10-year difference, red - above the 10-year difference, triangle - men, circle - women. The X-axis is the predicted age, and the Y-axis is the real age. Violin plot of SHAP values for each predictor in cohort C (B panel), cohort D (D panel).

**Figure 6.**
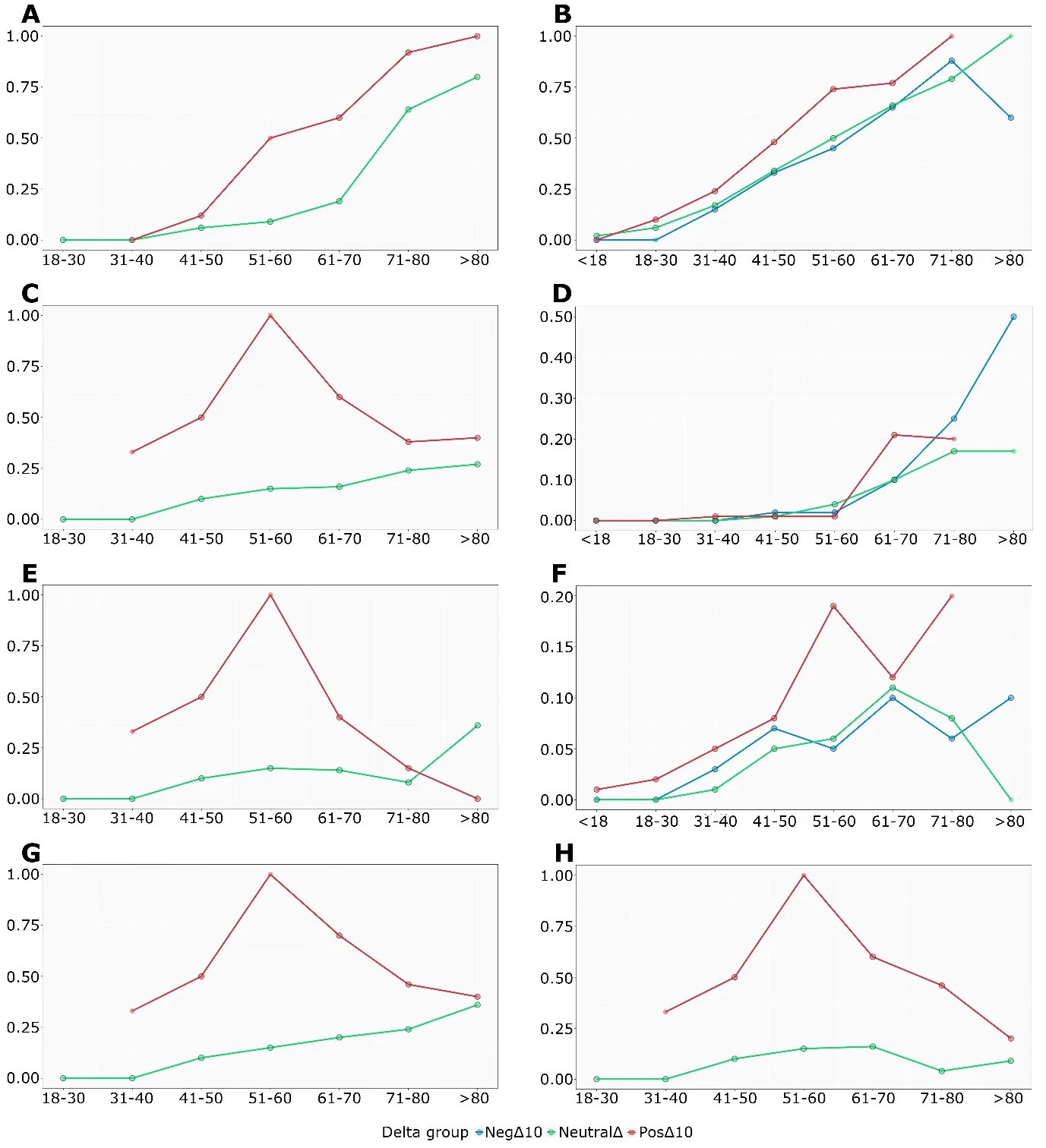
**Cardiovascular diseases frequency in Δ groups for age groups**. (**A**) I10-I15 Hypertensive diseases in Cohort A+C. (**B**) I10-I15 Hypertensive diseases in Cohort B+D. (**C**) I20-I25 Ischemic heart diseases in Cohort A+C. (**D**) I20-I25 Ischemic heart diseases in Cohort B+D. (**E**) I44-I45 Atrioventricular and left bundle-branch block, Other conduction disorders in Cohort A+C. (**F**) I44 Atrioventricular and left bundle-branch block in Cohort B+D. (**G**) I49.4 Extrasystoles in Cohort A+C. (**H**) I67 Other cerebrovascular diseases in Cohort A+C. Red - PosΔ10 cases, blue - NegΔ10, green - NeutralΔ. X-axis - age group, Y-axis - disease frequency.

Cardiovascular diseases. ICD10: I10-I15 - Hypertensive diseases and I20-I25 - ischemic heart diseases. We can note a persistent association of accelerated heart aging with the presence of pathology in most age groups. However, three changes are significant in 41-50, 51-60, 61-70 age groups (Table 5, Fig. 6A-D, and Supplementary Table 3-1). ICD10: I44-I45 - Atrioventricular and left bundle-branch block, other conduction disorders. Here we can note the association of accelerated aging of the heart with the presence of pathology also in most age groups, however, the changes are only reliable: 41-50, 51-60 (Table 5, Fig. 6E-F, Supplementary Table 3-1). ICD10: I49.4 - Extrasystoles and I67 - Other cerebrovascular diseases. There is an association of accelerated aging of the heart with the presence of hypertension in most age groups, however, the following are significant: 41-50, 51-60, 61-70, 71-80 (Table 5, Fig. 6G-H, Supplementary Table 3-1).

**Table 5:** The relationship of cardiovascular pathologies with accelerated heart aging in different age groups.

ICD-10	Description	Significant ↑ PosΔ10 freq. in age groups
**I10-I15**	Hypertensive diseases	41-50, 51-60, 61-70
**I20-I25**	Ischemic heart diseases	41-50, 51-60, 61-70
**I44-I45**	Atrioventricular and left bundle-branch block, Other conduction disorders	41-50, 51-60
**I49.4**	Extrasystoles	41-50, 51-60, 61-70
**I67**	Other cerebrovascular diseases	41-50, 51-60, 61-70, 71-80

**Figure 7.**
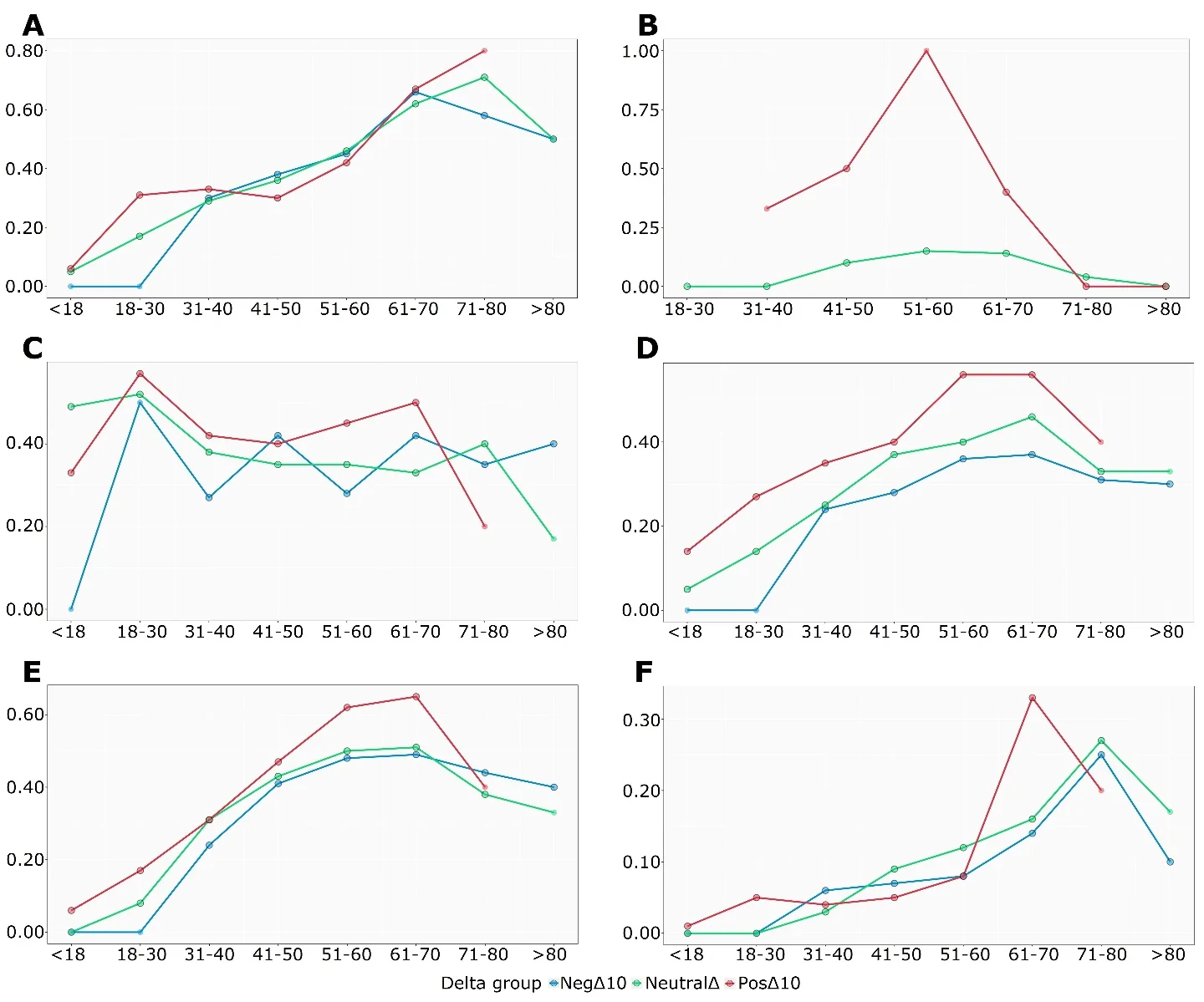
**Endocrine, metabolic and digestive disorders frequency in Δ groups for age groups**. (**A**) E00-E07 Disorders of thyroid gland in Cohort B+D. (**B**) E10-E14 Diabetes mellitus in Cohort A+C. (**C**) E55 Vitamin D deficiency in Cohort B+D. (**D**) E66 Obesity in Cohort B+D. (**E**) K76 Other diseases of liver in Cohort B+D. (**F**) K80 Cholelithiasis in Cohort B+D. Red - PosΔ10 cases, blue - NegΔ10, green - NeutralΔ. X-axis - age group, Y-axis - disease frequency.

Endocrine and metabolic disorders. ICD10: E00-E07 Disorders of the thyroid gland are associated with accelerated aging of the heart only in group 18-30, and E10-E14 Diabetes mellitus - 41-50, 51-60 (Table 6, Figure 7A-B, Supplementary Table 3-2). ICD10: E55 - Vitamin D deficiency is mostly associated with accelerated aging of the heart for ages 51-60, 61-70, while E66 - Obesity is typical for almost all ages (Table 6, Fig.7B-D, Supplementary Table 3-2). ICD-10: K76 - other liver diseases and K80 - cholelithiasis are associated with accelerated aging of the heart for ages 51-60, 61-70, however, a slight enrichment can be noticed for young people (Table 6, Fig. 7E-F, Supplementary Table 3-2).

**Figure 8.**
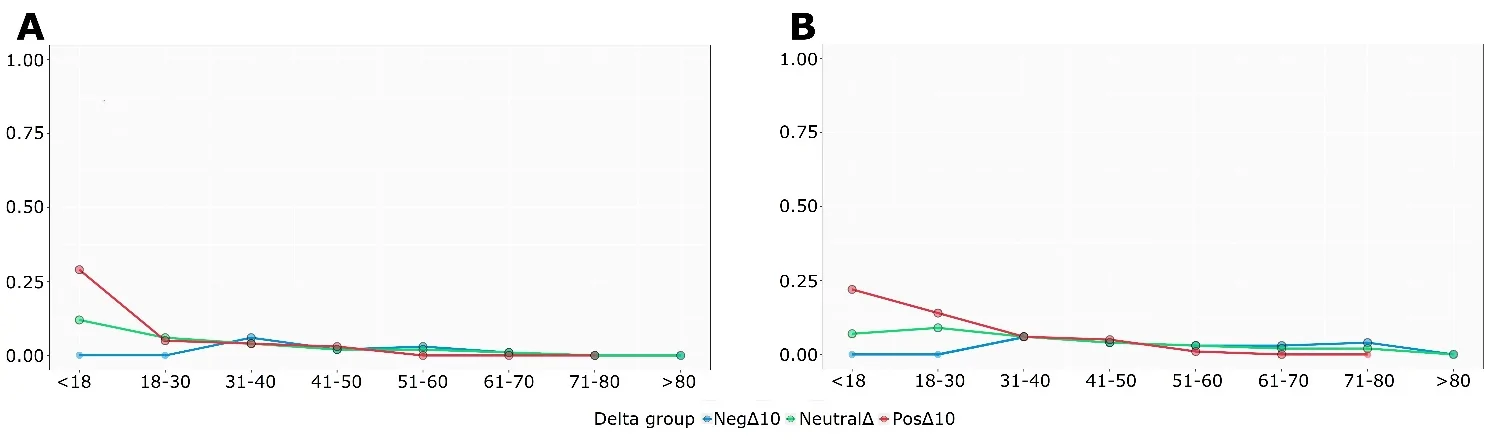
**Disease frequency in Δ groups for children (<18 years)**. (**A**) Q20 - Congenital malformations of cardiac chambers and connections in Cohort B+D. (**B**) L20-L30 - Dermatitis and eczema in Cohort B+D. Red - PosΔ10 cases, blue - NegΔ10, green - NeutralΔ. X-axis - age group, Y-axis - disease frequency.

Since there are children in the PosΔ10 group among the results, but they do not have age-related diseases, we examined them separately for different pathologies. For this age group, comparing PosΔ10 with NeutralΔ cases showed the greatest differences: L20-30 - dermatitis and eczema; and Q20 - congenital malformations of the heart chambers and connections (Fig.8, Supplementary Table 3-3).

**Table 6:** The relationship of endocrine and metabolic disorders with accelerated heart aging in different age groups.

ICD-10	Description	↑ Freq in Pos Δ 10 for age groups
**E00-E07**	Disorders of thyroid gland	18-30
**E10-E14**	Diabetes mellitus	41-50, 51-60
**E55**	Vitamin D deficiency	51-60, 61-70
**E66**	Obesity	18-30, 31-40, 41-50, 51-60, 61-70
**K76**	Other diseases of liver	51-60, 61-70
**K80**	Cholelithiasis	61-70

**Figure 9.**
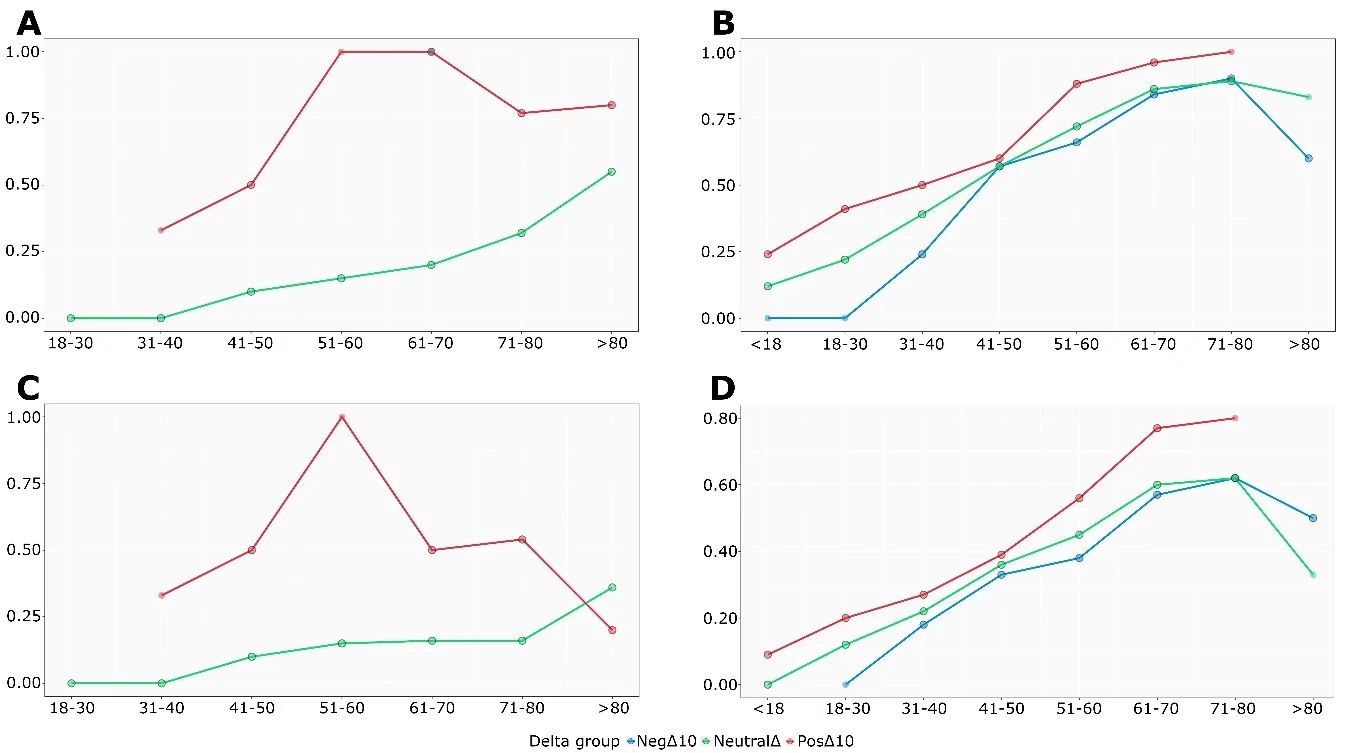
**Multimorbidity frequency in Δ groups for age groups**. (**A**) Multimorbidity N2 in Cohort A+C. (**B**) Multimorbidity N2 in Cohort B+D. (**C**) Multimorbidity N3 in Cohort A+C. (**D**) Multimorbidity N3 in Cohort B+D. Red - PosΔ10 cases, blue - NegΔ10, green - NeutralΔ. X-axis - age group, Y-axis - disease frequency.

Multimorbidity. When considering multimorbidity, we focused primarily on cases with at least two of the above chronic diseases (N2). We also looked at combinations of three (N3) and four diseases (N4). Multimorbidity N2 and N3 is associated with accelerated heart aging in most age groups, and, N2 can even occur in young persons, but N4 is more common in patients aged 41-70 (Table 7, Fig. 9, Supplementary Table 3-3).

**Table 7:** The dependence of multimorbidity on accelerated heart aging in different age groups.

ID	Description	↑ Freq in Pos Δ 10 for age groups
**N2**	At least 2 disorders	18-30, 31-40, 41-50, 51-60, 61-70, 71-80
**N3**	At least 3 disorders	41-50, 51-60, 61-70, 71-80
**N4**	At least 4 disorders	41-50, 51-60, 61-70

**Figure 10.**
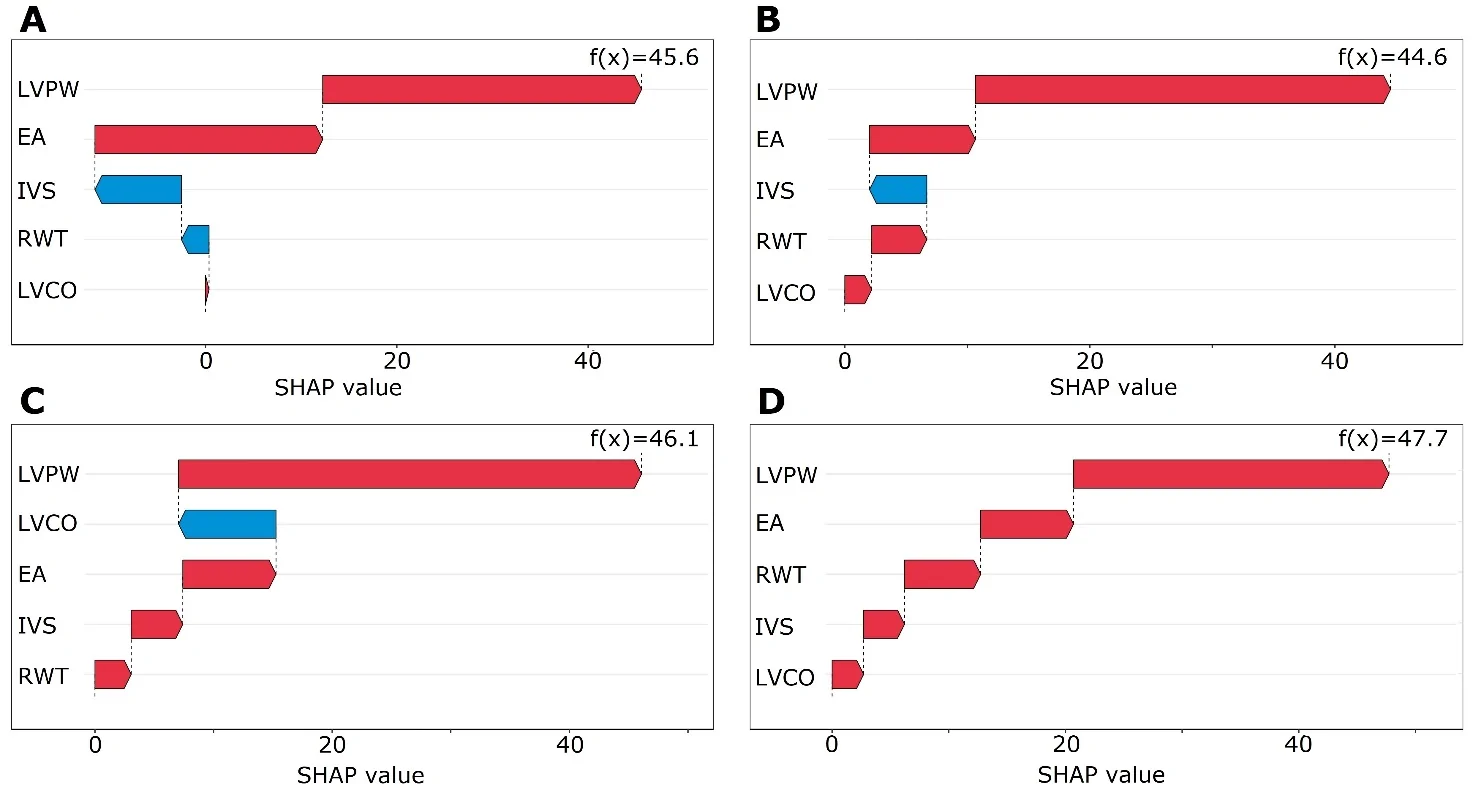
**Predictors SHAP values in four progeria cases**. (**A**) Progeria case 1. (**B**) Progeria case 2. (**C**) Progeria case 3. (**D**) Progeria case 4. X-axis - SHAP value, Y-axis - predictors. LVCO - cardiac output, L/minute; EA - E/A ratio of maximum flow rates in the first and second phases; RWT - relative wall thickness; IVS - thickness of the interventricular septum, cm; LVPW - thickness of the posterior left ventricular wall, cm.

Progeria. To understand the capabilities of our model, we ran data on four patients diagnosed with progeria, whose age varies from 4 to 8 years. The EchoAGE model estimated their echocardiographic parameters as comparable to those of people aged 45-50 years (Table 8, Fig.10).

Diachronic data. Part of the Cohorts B+D patients had repeated echocardiographic evaluation with an interval of several months to several years. In the diachronic analysis, we selected 240 patients with 3 to 6 (six is the maximum) visits. We compared their Δ groups with visits and found out that 105 patients were stably belonging to NeutralΔ and did not change the Δ group. Another nine patients were always in NegΔ10, and only three were stably in PosΔ10. At the same time, the most interesting cases are those who have changed the Δ group during visits. Therefore, for example, there are 47 patients whose condition worsened, that is, they belonged to NeutralΔ and moved to PosΔ10 (n=22) or were in NegΔ10 and moved to NeutralΔ (n=23) or passed through all three (n=2).

In addition, there were cases with the opposite effect. Patients were initially in the NeutralΔ group, and then they moved to NegΔ10 (n=58) or were in the PosΔ10 group but then moved to the NeutralΔ (n=17) group. Interestingly, patients who experienced a deterioration of Δ group - an increasing in their heart-aging rate - acquired the diagnoses discussed above. However, patients whose situation remained stable or improved (there was no acceleration in heart aging) did not tend to replenish with those diagnoses.

**Table 8:** Biological age for patients with progeria.

Progeria case	Sex	Age	Height	Predicted Age	Delta
**1**	Female	7	103	46	39
**2**	Female	4	89	45	41
**3**	Male	6	101	46	40
**4**	Male	8	100	48	40

## DISCUSSION

The current research focused on the comprehension of possibility to determine of the biological age based on echocardiographic data. We identified the most promising indicators as part of our work, built, and trained neural network models for both sexes. Our approach has a high predictive ability (MAE < 4, R^2^ ~ 0.9, rho > 0.9) and is unique using echocardiographic data to develop a neural network solution.

To date, there have been known approaches to determining biological age that involve various "markers" of human aging, including indicators of the cardiovascular system. In our recent work, we described the relationship between biological age and arterial health. We proposed a regression model based on data from duplex scanning of the carotid artery and applanation tonometry, with MAE of 6.91 for men and 5.87 for women [21]. A series of other studies have demonstrated sex-specific models, based on parameters such as systolic blood pressure [7], fasting blood sugar, hemoglobin, total cholesterol, triglycerides [10-11], serum creatinine, estimated glomerular filtration rate, and aspartate aminotransferase [12-13]. These models have an average correlation coefficient in the range of 0.50 to 0.75.

Another study focused on the assessment of heart rate. It was shown that the resting heart rate does not depend on age. However, the maximum change in heart rate has the highest negative correlation with age (r = -0.67) [9].

The D'Agostino research group created a cardiometabolic age formula based on systolic blood pressure, diabetes, body mass index, hypertension treatment, general indicators of age, gender, and smoking [22].

Another series of works that used neural network or regression models were based on electrocardiographic data, with R2 of approximately 0.79 [4; 8].

In addition, there is a model for estimating the biological age of the heart based on magnetic resonance imaging measurement of the shape of the ventricles and the structure of the myocardium, with a MAE of approximately 4.95 to 5.48, and an R^2^ value of 0.3. Within this study, positive correlations were found between the difference in heart age and various indicators of obesity. A larger delta in heart aging was associated with higher triglycerides, higher low-density lipoprotein cholesterol levels, and lower levels of high-density lipoproteins [23].

Moreover, there are only a few studies devoted to analyzing echocardiographic indicators, although they do not offer a predictive model. They demonstrate the presence of age-related changes in the rate of myocardial movement and also identify at-risk groups associated with pathological conditions such as hypertension [24-25].

Besides attempts to determine the biological age of the heart, there are known attempts to create models of the age of the kidneys, lungs, pancreas or liver [30]. Herewith the authors noted that the heart age model still had the greatest predictive power compared to the rest of the listed ones (R2 of the order of 0.72 versus 0.57, 0.44, 0.32 and 0.13, respectively), most of these models held better in women than men [26].

As for the advantages of our research and the developed model for determining the biological age, we can note that to date, this is the only calculator (DNN) for estimating the biological age of the heart based on echocardiography. Furthermore, we have demonstrated the relationship between the age assessments carried out by our model and age-related diseases and have confirmed the adequacy of this assessment using data from multimorbidity patients and children with Progeria. The undeniable advantage of this approach is the lack of invasiveness of the method. The model has a high level of accuracy, with a wide range of possible assessments (patients without any age-related diseases ranging from 4 to 91 years were tested). Furthermore, the reliability of the assessment is based on repeated/diachronic data, which provides a persistent reproducible result.

However, our approach has its own difficulties and limitations that need to be mentioned. One of the difficulties in obtaining an assessment according to our protocol is the need for instrumental diagnostics, such as echocardiography, which is not included in standard screenings and is carried out mainly according to specific indications. Additionally, inaccuracies may be allowed in recording echocardiogram results, which may depend on the experience of a doctor and, accordingly, may lead to the distortion of data processing. Among the limitations, it is important to note that all analytics in our work were performed using data from echocardiography obtained exclusively from patients of the Caucasian patients. Therefore, possible population biases were not considered.

In conclusion, this work confirmed the possibility of estimating human biological age based on echocardiographic data. We created a highly accurate neural network model to evaluate the biological age of the heart and detect changes in data related to age-related diseases.

## Supplementary Materials

The Supplementary data can be found online at: www.aginganddisease.org/EN/10.14336/AD.2024.0615.
