# Supplementary material for: EchoAGE: Echocardiography-based Neural Network Model Forecasting Heart Biological Age
Source: Aging Dis. 2024 Jul 28;16(4):2383–97. doi: 10.14336/AD.2024.0615 (PMC12221396; doi:10.14336/AD.2024.0615)
Supplement: Supplementary file 1 — The Supplementary data can be found online at: www.aginganddisease.org/EN/10.14336/AD.2024.0615. [file AD-16-4-2383-s.pdf]

SUPPLEMENTARY DATA

EchoAGE: Echocardiography-based Neural Network model forecasting Heart Biological Age

Anastasia A. Kobelyatskaya, Zulfiya G. Guvatova, Olga N. Tkacheva, Fedor I. Isaev, Anastasiia L. Kungurtseva, Alisa V. Vitebskaya, Anna V. Kudryavtseva, Ekaterina V. Plokhova, Lubov V. Machekhina, Irina D. Strazhesko, Alexey A. Moskalev

© 2024. Kobelyatskaya AA. et al. Published online at http://www.aginganddisease.org/EN/10.14336/AD.2024.0615

SUPPLEMENTARY DATA

Supplementary Table 1. Correlations of potential predictors.

Description	Unit	Is factor variable	Man-datory or calcu-lated	ID	% evaluated cases (from cohort)	Rho	Rho _abs	p-value	Rho Male	Rho _abs Male	p-value Male	Rho Female	Rho _abs Female	p-value Female	Max of all abs rho
relative thickness of the walls of the left ventricular	ratio	0	1	RWT	100	0.77	0.77	7.6E-51	0.76	0.76	7.9E-16	0.81	0.81	5.1E-40	0.81
Cardiac Output	L/minute	0	1	LV_CO	100	-0.74	0.74	1.5E-44	-0.71	0.71	2.6E-13	-0.72	0.72	9.1E-29	0.74
ratio peak velocities A and E waves	ratio	0	1	E_A	100	-0.68	0.68	5.6E-35	-0.64	0.64	2.7E-10	-0.73	0.73	1.1E-29	0.73
thickness of interventricular septum	cm	0	1	IVS	100	0.66	0.66	4.2E-32	0.54	0.54	2.4E-07	0.70	0.70	2.5E-26	0.70
posterior wall thickness	cm	0	1	LVPW	100	0.62	0.62	2.0E-27	0.57	0.57	3.4E-08	0.64	0.64	1.8E-20	0.64
end-diastolic volume	ml	0	1	LV_EDV	100	-0.53	0.53	5.4E-19	-0.57	0.57	3.7E-08	-0.51	0.51	1.6E-12	0.57
end-systolic volume	ml	0	1	LV_ESV	100	-0.54	0.54	9.9E-30	-0.53	0.53	5.0E-10	-0.53	0.53	7.7E-20	0.54
end-systolic left ventricular diameter	cm	0	1	LVSD	100	-0.36	0.36	5.1E-09	-0.54	0.54	3.5E-07	-0.35	0.35	3.5E-06	0.54
ejection fraction	%	0	1	LV_EF	100	0.49	0.49	3.5E-16	0.41	0.41	1.7E-04	0.52	0.52	2.4E-13	0.52
aorta diameter	cm	0	1	AO_D	100	0.35	0.35	1.2E-08	0.18	0.18	1.0E-01	0.44	0.44	2.1E-09	0.44
end-diastolic left ventricular diameter	cm	0	1	LVDD	100	-0.22	0.22	5.7E-04	-0.43	0.43	8.6E-05	-0.17	0.17	2.5E-02	0.43
E wave peak	cm/s	0	1	E_P	100	-0.35	0.35	1.3E-08	-0.42	0.42	9.8E-05	-0.36	0.36	1.4E-06	0.42

© 2024. Kobelyatskaya AA. et al. Published online at http://www.aginganddisease.org/EN/10.14336/AD.2024.0615

SUPPLEMENTARY DATA

fractional shortening	%	0	1	FS	100	0.33	0.33	1.5E-07	0.18	0.18	1.0E-01	0.41	0.41	3.3E-08	0.41
stroke volume	ml	0	1	LV_SV	100	-0.40	0.40	6.8E-11	-0.39	0.39	3.3E-04	-0.39	0.39	2.0E-07	0.40
left atrial volume index	ml/m2	0	1	LAVI	100	0.28	0.28	8.9E-06	0.11	0.11	3.3E-01	0.38	0.38	3.8E-07	0.38
left ventricular mass index	g/m2	0	1	MMI	100	0.23	0.23	2.6E-04	0.26	0.26	2.2E-02	0.26	0.26	5.8E-04	0.26
RWT > 0,42	(0 - no; 1 - yes)	1	1	H_RWT	100	0.75	0.75	2.3E-45	0.70	0.70	1.1E-12	0.78	0.78	1.6E-36	0.78
E/A < 1,0	(0 - no; 1 - yes)	1	1	L_E_A	100	0.62	0.62	1.8E-27	0.57	0.57	3.2E-08	0.67	0.67	3.2E-23	0.67
H_RWT = 1 & H_MMI = 0	(0 - no; 1 - yes)	1	1	CRLV	100	0.55	0.55	7.7E-21	0.48	0.48	8.9E-06	0.57	0.57	4.1E-16	0.57
IVS > 1,1	(0 - no; 1 - yes)	1	1	H_IVS	100	0.40	0.40	6.1E-11	0.40	0.40	2.3E-04	0.49	0.49	1.1E-11	0.49
H_RWT = 1 & H_MMI = 1	(0 - no; 1 - yes)	1	1	CHLV	100	0.33	0.33	1.1E-07	0.37	0.37	9.3E-04	0.34	0.34	4.4E-06	0.37
LAVI > 28	(0 - no; 1 - yes)	1	1	H_LAVI	100	0.20	0.20	2.0E-03	0.05	0.05	6.9E-01	0.32	0.32	2.8E-05	0.32
MMI > 102 male, MMI > 88 female	(0 - no; 1 - yes)	1	1	H_MMI	100	0.21	0.21	9.6E-04	0.20	0.20	8.3E-02	0.23	0.23	2.8E-03	0.23
LVPW > 1,1	(0 - no; 1 - yes)	1	1	H_LVPW	100	0.11	0.11	9.1E-02	0.04	0.04	7.4E-01	0.20	0.20	9.7E-03	0.20
H_RWT = 0 & H_MMI = 1	(0 - no; 1 - yes)	1	1	ERLV	100	-0.09	0.09	1.5E-01	-0.17	0.17	1.4E-01	-0.07	0.07	3.4E-01	0.17
AO_D > 3,8	(0 - no; 1 - yes)	1	1	H_AO_D	100	0.06	0.06	3.3E-01	0.08	0.08	5.1E-01	0.13	0.13	1.1E-01	0.13
Calcium index	ratio	0	0	CAIND	4	0.74	0.74	5.4E-02				0.85	0.85	5.4E-02	0.85

© 2024. Kobelyatskaya AA. et al. Published online at http://www.aginganddisease.org/EN/10.14336/AD.2024.0615

SUPPLEMENTARY DATA

Isovolumic relaxation time	millisecond	0	0	IVRT	100	0.68	0.68	1.5E-35	0.66	0.66	3.1E-11	0.74	0.74	4.5E-30	0.74
Pulm S/D	ratio	0	0	S_D	100	0.72	0.72	7.4E-41	0.67	0.67	2.3E-11	0.69	0.69	4.2E-25	0.72
E'/A' lateral	ratio	0	0	E1_A1	100	-0.67	0.67	4.2E-34	-0.68	0.68	4.0E-12	-0.71	0.71	6.0E-27	0.71
E' lateral	cm/s	0	0	E1	100	-0.60	0.60	3.7E-25	-0.60	0.60	3.8E-09	-0.64	0.64	8.2E-21	0.64
Deceleration time	millisecond	0	0	DT	100	0.52	0.52	1.8E-18	0.48	0.48	6.7E-06	0.59	0.59	6.3E-17	0.59
A wave peak	cm/s	0	0	A_P	100	0.52	0.52	7.1E-19	0.45	0.45	2.7E-05	0.55	0.55	1.2E-14	0.55
A reversal duration	millisecond	0	0	ARD	100	0.44	0.44	2.6E-13	0.50	0.50	3.2E-06	0.48	0.48	6.4E-11	0.50
A reversal velocity	cm/s	0	0	ARV	100	0.41	0.41	1.5E-11	0.35	0.35	1.7E-03	0.47	0.47	1.4E-10	0.47
E/E' lateral	ratio	0	0	E_E1	100	0.41	0.41	3.2E-11	0.37	0.37	8.3E-04	0.42	0.42	1.1E-08	0.42
S' lateral	cm/s	0	0	S1	100	-0.35	0.35	1.1E-08	-0.25	0.25	2.4E-02	-0.39	0.39	1.3E-07	0.39
A' lateral	cm/s	0	0	A1	100	0.31	0.31	4.4E-07	0.20	0.20	7.1E-02	0.36	0.36	1.8E-06	0.36
S/D > 1	(0 - no; 1 - yes)	1	0	H_S_D	100	0.68	0.68	1.8E-34	0.66	0.66	4.1E-11	0.65	0.65	2.6E-21	0.68
L_E_A = 1 or L_E1_A1 = 1	(0 - no; 1 - yes)	1	0	DD_LV	100	0.62	0.62	3.0E-27	0.57	0.57	5.4E-08	0.67	0.67	4.9E-23	0.67
E'/A' < 1	(0 - no; 1 - yes)	1	0	L_E1_A1	100	0.59	0.59	6.5E-25	0.56	0.56	7.3E-08	0.64	0.64	9.2E-21	0.64
IVRT > 90	(0 - no; 1 - yes)	1	0	H_IVRT	100	0.55	0.55	1.3E-20	0.49	0.49	4.7E-06	0.60	0.60	3.3E-18	0.60

© 2024. Kobelyatskaya AA. et al. Published online at http://www.aginganddisease.org/EN/10.14336/AD.2024.0615

SUPPLEMENTARY DATA

DT > 220	(0 - no; 1 - yes)	1	0	H_DT	100	0.42	0.42	5.2E-12	0.47	0.47	1.0E-05	0.44	0.44	2.0E-09	0.47
E1 < 8	(0 - no; 1 - yes)	1	0	L_E1	100	0.36	0.36	5.2E-09	0.36	0.36	1.0E-03	0.39	0.39	1.2E-07	0.39
ARV > 35	(0 - no; 1 - yes)	1	0	H_ARV	100	0.28	0.28	6.5E-06	0.19	0.19	9.3E-02	0.36	0.36	1.8E-06	0.36

Supplementary Table 2. Quality indicators of models.

		train rates				test rates				mean rates				scales mean rates				
model №	predictors	mse	mae	mape	rmse	mse	mae	mape	rmse	mse	mae	mape	rmse	mse	mae	mape	rmse	score
1	LVCO_EA_RWT_IVS	49.79	5.41	12.03	7.06	44.65	5.31	11.85	6.68	47.22	5.36	11.94	6.87	0.225	0.257	0.197	0.214	0.894
2	LVCO_EA_RWT_LVPW	41.91	5.14	11.37	6.47	46.21	5.59	12.25	6.80	44.06	5.36	11.81	6.64	0.277	0.256	0.206	0.241	0.980
3	LVCO_EA_IVS_LVPW	59.39	6.11	13.95	7.71	60.52	6.30	13.80	7.78	59.96	6.21	13.87	7.74	0.016	0.139	0.067	0.114	0.337
4	LVCO_RWT_IVS_LVPW	49.94	5.60	12.55	7.07	51.25	5.58	12.53	7.16	50.59	5.59	12.54	7.11	0.170	0.224	0.157	0.186	0.738
5	EA_RWT_IVS_LVPW	59.72	6.25	14.14	7.73	47.66	5.37	11.38	6.90	53.69	5.81	12.76	7.32	0.119	0.194	0.142	0.163	0.619
6	LVCO_EA_RWT_IVS_LVPW	37.69	4.86	10.59	6.14	50.09	5.72	12.42	7.08	43.89	5.29	11.51	6.61	0.280	0.266	0.226	0.244	1.017
									maxs	60.96	7.21	14.87	8.74					

Supplementary Table 3. Frequency analysis of conditions.

1 – Cardio

ID	Age group	Chi-squared p-value: across 3 delta groups	Chi-squared p-value: PosΔ10 vs NegΔ10	Chi-squared p-value: PosΔ10 vs NeutralΔ	Frequency difference: PosΔ10 vs NegΔ10	Frequency difference: PosΔ10 vs NeutralΔ	Cohorts
I10-I15	41-50	0.01583	0.03243	0.00612	0.15	0.14	BD
I10-I15	51-60	0.00008	0.00003	0.00015	0.29	0.24	BD
I10-I15	61-70	NA	NA	0.01562	NA	0.41	AC
I20-I25	41-50	NA	NA	0.01389	NA	0.40	AC
I20-I25	51-60	NA	NA	0.02938	NA	0.85	AC
I20-I25	61-70	NA	NA	0.00529	NA	0.44	AC
I20-I25	61-70	0.04744	0.04977	0.03024	0.11	0.11	BD
I44-I45	41-50	NA	NA	0.01389	NA	0.40	AC
I44-I45	51-60	NA	NA	0.02938	NA	0.85	AC
I44-I45	51-60	0.00003	0.00023	0.00005	0.14	0.13	BD

© 2024. Kobelyatskaya AA. et al. Published online at http://www.aginganddisease.org/EN/10.14336/AD.2024.0615

I49.4	41-50	NA	NA	0.01389	NA	0.40	AC
I49.4	51-60	NA	NA	0.02938	NA	0.85	AC
I49.4	61-70	NA	NA	0.00291	NA	0.50	AC
I67	41-50	NA	NA	0.01389	NA	0.40	AC
I67	51-60	NA	NA	0.02938	NA	0.85	AC
I67	61-70	NA	NA	0.00529	NA	0.44	AC
I67	71-80	NA	NA	0.00616	NA	0.42	AC

2 - Endocr., Metabol., Digest.

ID	Age group	Chi-squared p-value			Frequency difference		Cohorts
		across 3 delta groups	PosΔ10 vs NegΔ10	PosΔ10 vs NeutralΔ	PosΔ10 vs NegΔ10	PosΔ10 vs NeutralΔ	
E00-E07	18-30	0.08935	0.87600	0.06241	0.31	0.14	BD
E10-E14	41-50	NA	NA	0.01389	NA	0.40	AC
E10-E14	51-60	NA	NA	0.02938	NA	0.85	AC
E55	51-60	0.01720	0.01031	0.11223	0.17	0.10	BD
E55	61-70	0.01297	0.36127	0.03085	0.08	0.17	BD
E66	18-30	0.07199	0.95701	0.04756	0.27	0.13	BD
E66	31-40	0.03685	0.30410	0.01507	0.11	0.10	BD
E66	41-50	0.09642	0.07453	0.59468	0.12	0.03	BD
E66	51-60	0.00726	0.00266	0.01241	0.20	0.16	BD
E66	61-70	0.01697	0.02346	0.23356	0.19	0.10	BD
K76	51-60	0.11114	0.05531	0.06331	0.14	0.12	BD
K76	61-70	0.13436	0.06501	0.11067	0.16	0.14	BD
K80	61-70	0.00397	0.00306	0.00360	0.19	0.17	BD

3 – Children

ID	Age group	Chi-squared p-value			Frequency difference		Cohorts
		across 3 delta groups	PosΔ10 vs NegΔ10	PosΔ10 vs NeutralΔ	PosΔ10 vs NegΔ10	PosΔ10 vs NeutralΔ	
L20-L30	<18	NA	NA	0.07493	NA	0.15	BD
Q20	<18	NA	NA	0.06611	NA	0.17	BD

4 - Multimorbid.

ID	Age group	Chi-squared p-value			Frequency difference		Cohorts
		across 3 delta groups	PosΔ10 vs NegΔ10	PosΔ10 vs NeutralΔ	PosΔ10 vs NegΔ10	PosΔ10 vs NeutralΔ	
n2	18-30	0.00106	0.53451	0.00071	0.48	0.26	BD
n2	31-40	0.02172	0.01838	0.07339	0.24	0.08	BD
n2	41-50	NA	NA	0.01389	NA	0.40	AC
n2	51-60	NA	NA	0.02938	NA	0.85	AC
n2	51-60	0.00671	0.00260	0.01104	0.19	0.15	BD
n2	61-70	NA	NA	0.00000	NA	0.80	AC
n2	61-70	0.05312	0.02813	0.03030	0.14	0.13	BD

© 2024. Kobelyatskaya AA. et al. Published online at http://www.aginganddisease.org/EN/10.14336/AD.2024.0615

SUPPLEMENTARY DATA

n2	71-80	NA	NA	0.02209	NA	0.45	AC
n3	41-50	NA	NA	0.01389	NA	0.40	AC
n3	51-60	NA	NA	0.02938	NA	0.85	AC
n3	51-60	0.01727	0.01014	0.09454	0.18	0.11	BD
n3	61-70	NA	NA	0.03461	NA	0.34	AC
n3	61-70	0.03613	0.01569	0.03167	0.20	0.17	BD
n3	71-80	NA	NA	0.03906	NA	0.38	AC
n4	41-50	NA	NA	0.01389	NA	0.40	AC
n4	51-60	NA	NA	0.02938	NA	0.85	AC
n4	51-60	0.01941	0.00836	0.06264	0.15	0.10	BD
n4	61-70	0.00169	0.00181	0.00077	0.25	0.25	BD

© 2024. Kobelyatskaya AA. et al. Published online at http://www.aginganddisease.org/EN/10.14336/AD.2024.0615
